# Visualization of Type IV Pili: Linking Structural Architecture, Dynamic Function, and Translational Opportunities

**DOI:** 10.3390/biology15100758

**Published:** 2026-05-09

**Authors:** Jingchao Zhang, Yutong Liu

**Affiliations:** 1 College of Ecology and Environment, Chengdu University of Technology, Chengdu 610059, China; 2 Institute of Fundamental and Frontier Sciences, University of Electronic Science and Technology of China, Chengdu 611731, China; murphytong@std.uestc.edu.cn

**Keywords:** biofilm formation, cryo-EM, twitching motility, type IV pili, visualization techniques

## Abstract

Type IV pili are widespread and multifunctional filamentous nanomachines that contribute to bacterial motility, adhesion, surface sensing, DNA uptake, biofilm formation, and, in some organisms, extracellular electron transfer. Owing to their small diameter, rapid dynamics, and sensitivity to experimental perturbation, type IV pili have historically been difficult to visualize in their native states. Recent advances in cryo-electron microscopy (cryo-EM), cryo-electron tomography (cryo-ET), fluorescence-based live-cell imaging, and label-free approaches such as interferometric scattering microscopy (iSCAT) have substantially expanded our ability to observe type IV pili across spatial and temporal scales. In this review, we summarize how these visualization strategies have reshaped current understanding of type IV pili, from conserved filament architecture and envelope-spanning assembly machineries to force-dependent behaviors and context-specific physiological functions. We further discuss how imaging-informed knowledge may support translational efforts, including antivirulence intervention, vaccine design, bioelectronic optimization, and microbial engineering, while emphasizing the current limitations of these applications. By integrating structural, dynamic, and functional perspectives, this review aims to provide a coherent framework for future studies of type IV pili in microbiology, infection biology, and biotechnology.

## Simple Summary

Type IV pili are dynamic bacterial filaments involved in motility, adhesion, surface sensing, biofilm formation, and DNA uptake. This review highlights how advanced imaging methods—cryo-EM, cryo-ET, live-cell fluorescence, iSCAT, and force-based assays—have revealed type IV pili architecture, dynamics, and functions across scales. It also discusses translational potential in antivirulence strategies, vaccine-related antigen mapping, and microbial engineering, while noting current technical and biological limitations.

## 1. Introduction

Microorganisms rely on specialized surface appendages to interact with, respond to, and adapt to complex environments [1,2]. Among them, type IV pili, because of their dynamic extension–retraction cycle and broad functional repertoire, have become a major focus in microbiology, pathogen biology, and applied microbiology. They contribute to key physiological processes, including cell adhesion, host interaction, DNA uptake, biofilm formation, twitching motility, and, in some systems, extracellular electron transfer and environmental sensing [3,4,5]. Therefore, elucidating the structure and function of type IV pili is of great significance for understanding microbial environmental adaptation and host-associated behaviors, as well as for developing new anti-infective strategies.

Early understanding of type IV pili relied primarily on conventional light microscopy, negative-stain electron microscopy, and genetic inference [5,6]. Early visualization was constrained by the diffraction limit of conventional light microscopy, which, because individual pili are only a few nanometers in diameter, precluded their direct resolution. Consequently, their presence was initially inferred from surface-associated phenotypes such as twitching motility, adhesion, and natural transformation [4,7,8]. Electron microscopy subsequently revealed filamentous surface appendages. Negative-stain and transmission electron microscopy later provided direct visual evidence of thin filamentous appendages on bacterial surfaces and were instrumental in establishing the existence, morphology, and broad distribution of type IV pili across diverse microorganisms [5,6,9]. These early approaches contributed to recognition that type IV pili are dynamic rather than static appendages, but traditional fixation and negative-staining approaches often introduced structural distortion, and both methods were limited in their ability to capture rapid extension–retraction cycles under native physiological conditions [5,10].

Despite their foundational importance, conventional visualization approaches had important limitations [4,5]. Chemical fixation, dehydration, staining, and surface adsorption could distort filament morphology, obscure fragile structures, or alter pilus distribution on the cell surface [4,11]. More importantly, static imaging could not resolve the fast and reversible behaviors that are central to type IV pili function, including extension, retraction, transient surface attachment, and force-dependent remodeling [9,12,13]. Cryo-EM and cryo-ET address these limitations by using rapid vitrification to preserve specimens in near-native states, permitting high-resolution characterization of pilus architecture and capturing structural snapshots of the assembly machinery in different functional states [14]. Complementing these structural methods, fluorescence-based imaging and label-free techniques such as iSCAT excel at capturing real-time dynamics in living cells [13]. These advances have strengthened attempts to relate filament architecture and machine organization to biological function.

At the same time, fluorescence-based imaging has provided powerful tools for probing type IV pili dynamics in living cells. Techniques such as total internal reflection fluorescence microscopy (TIRF) and targeted fluorescent labeling have enabled direct observation of pilus extension, retraction, turnover, and localization [12,15,16,17,18]. Reporter-based imaging has also made it possible to connect type IV pili activity with intracellular signaling states and surface-response pathways [13]. More recently, label-free methods such as interferometric scattering microscopy (iSCAT) have further reduced experimental perturbation and enabled high-speed visualization of fragile or transient pilus behaviors [13]. Together, these methods have transformed the field from one centered on morphological description to one capable of linking type IV pili architecture, dynamics, and function across spatial and temporal scales [4,9].

This review focuses on type IV pili and synthesizes key findings from recent visualization studies. It first summarizes major imaging strategies and their respective strengths and limitations. It then discusses how visualization has advanced current understanding of type IV pili structural organization and functional behaviors, including twitching motility, surface sensing, biofilm formation, and natural transformation. Finally, it evaluates the translational implications of these advances in medicine, environmental microbiology, and biotechnology, while highlighting unresolved questions and technical challenges. By organizing the literature along a technology–structure–function–application axis, we aim to provide a coherent framework for future type IV pili research.

## 2. Technological Advances in Type IV Pili Visualization

**Figure 1:**
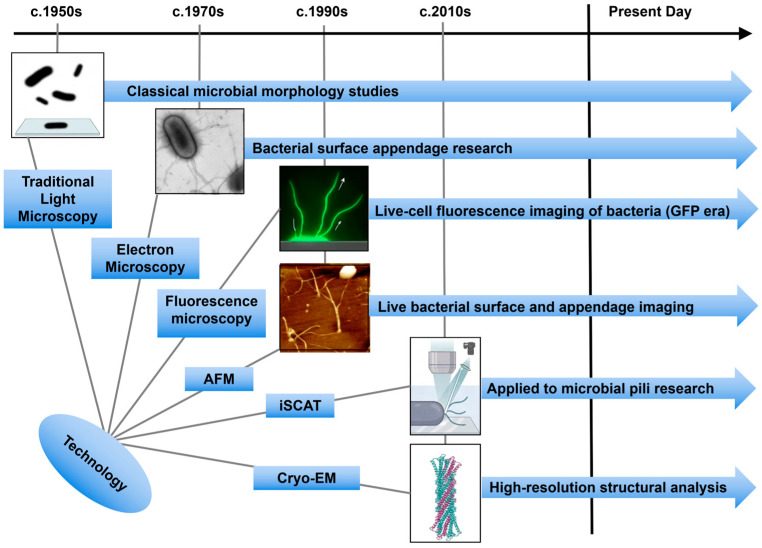
Timeline of imaging technologies used to study type IV pili. Traditional light microscopy enabled early observation of microbial morphology (c.1950s) [28], followed by electron microscopy for visualization of bacterial surface appendages (c.1970s). Fluorescence microscopy and AFM expanded live-cell and surface imaging of pili-associated structures and dynamics (c.1990s) [29]. More recently, iSCAT enabled label-free tracking of pili dynamics (c.2010s) [13], whereas cryo-EM has provided high-resolution structural insights into type IV pili architecture [30]. Together, these approaches link type IV pili structure, dynamics, and translational potential.

Throughout the history of type IV pili research, methodological innovation has been central to conceptual progress (Figure 1) [4,5,9]. Rather than replacing one another in a strictly linear manner, electron microscopy, fluorescence microscopy, cryo-EM/cryo-ET, iSCAT, and force-based methods now serve as complementary tools that address different aspects of type IV pili biology [4,9]. Electron microscopy remains valuable for morphological analysis, fluorescence-based imaging is essential for observing pilus behavior in living cells [7,11], cryo-EM and cryo-ET provide high-resolution structural information in near-native states [19,20,21], iSCAT enables rapid label-free tracking of thin surface filaments [13], and AFM or optical trapping approaches quantify force generation and adhesive mechanics [22,23]. The major challenge in the field is that type IV pili are thin (~5–8 nm in diameter), can extend for micrometers, and often undergo rapid and transient extension–retraction cycles, making them difficult to visualize with any single method [4,9,12]. As summarized in Figure 1, advances in visualization have progressively expanded the accessible spatial and temporal scales of type IV pili analysis, from early morphological observation by electron microscopy to live-cell fluorescence imaging, label-free detection by iSCAT, force measurement by AFM, and near-native structural analysis by cryo-EM and cryo-ET. This technological progression has fundamentally shifted the field from descriptive imaging of pili to an integrated understanding of their architecture, dynamics, and physiological functions. These techniques differ not only in resolution but also in what kind of question they can answer [4,9]. Cryo-EM resolves purified filaments or complexes at near-atomic resolution and is especially powerful for defining pilin packing, helical symmetry, and surface-exposed features [9,19,24]. Cryo-ET, by contrast, visualizes macromolecular assemblies in situ and is therefore better suited for understanding how the type IV pili machine is organized across the cell envelope [20,21,25]. Fluorescence microscopy enables live-cell analysis of pilus dynamics, localization, and regulatory coupling, although it typically requires labeling strategies that may perturb native function [12,15,26]. iSCAT detects unlabeled filaments through interference between scattered and reflected light and is particularly useful for rapid, minimally perturbative observation of pilus extension, attachment, and retraction [13]. AFM and related force-based approaches provide complementary mechanical information by measuring adhesion, rupture force, elasticity, or load-dependent behaviors that cannot be inferred from imaging alone [9,22]. Early research was constrained by the ~200 nm resolution limit of light microscopy, which precluded the detailed visualization of motile organelles like type IV pili. While electron microscopy, including negative staining approaches, was instrumental in first confirming the dynamic nature of type IV pili, these early techniques had important limitations in preserving native filament architecture and failed to capture rapid extension–retraction processes in real time in living cells [5,10,27].

In 2001, Skerker and Berg pioneered live-cell imaging of type IV pili by labeling non-flagellated *Pseudomonas aeruginosa* with Cy3 and imaging cells by total internal reflection fluorescence microscopy (TIRFM), directly observing repeated cycles of pilus extension and retraction at estimated rates of approximately 0.5 μm/s and describing pilus bending behavior consistent with Brownian motion [12]. This landmark study established a direct mechanistic link between pilus dynamics and twitching motility, laying the foundation for mechanistic investigation of type IV pili dynamics in living cells [12]. Subsequently, more specific labeling strategies were developed. Ellison et al. introduced a versatile approach based on cysteine substitution in the major pilin, followed by conjugation with thiol-reactive maleimide fluorophores, enabling live-cell labeling of type IV pili in multiple bacterial species with improved specificity [18,31]. Zhang et al. further developed a plasmid-inducible pilin replacement system that allowed switchable fluorescent labeling without permanent genome editing and enabled analysis of pilin pool replenishment dynamics in *Pseudomonas aeruginosa* [32]. Inducible pilin-replacement strategies have allowed controlled incorporation of fluorescently traceable pilin subunits while partially mitigating the functional limitations associated with permanent chromosomal modification [32]. Although these approaches substantially improved imaging specificity, they still require caution because exogenous labeling may alter pilus assembly, filament mechanics, or native function. Even so, fluorescence-based studies must be interpreted cautiously because fluorophore attachment, incomplete labeling, altered pilin stoichiometry, or condition-dependent pilus expression can influence filament assembly, mechanics, and turnover [4,26].

To minimize perturbation introduced by exogenous tags, label-free imaging techniques have gained increasing prominence. Among these, interferometric scattering microscopy (iSCAT) is particularly notable for its high spatial and temporal resolution. Talà et al. first applied iSCAT to *Pseudomonas aeruginosa* to monitor type IV pili extension, attachment, and retraction in intact living cells with millisecond time resolution [13]. This work revealed short-lived pilus–surface interactions and suggested that bacteria can rapidly retract pili following substrate contact. Subsequent studies further showed that *Pseudomonas aeruginosa* coordinates type IV pili attachment and retraction through temporally controlled activation of the retraction machinery, supporting a mechanosensory model of early surface engagement [13]. Additionally, iSCAT has been applied to the archaeon *Haloferax volcanii*, demonstrating that TFP is crucial for the mechanical integrity of biofilms under fluid shear stress [33].

Atomic force microscopy (AFM), which combines picoNewton-scale force sensitivity with operation in liquid environments, complements optical imaging by quantifying the adhesive and mechanical properties of type IV pili under hydrated conditions [9,22]. Touhami et al. measured rupture forces of single *Pseudomonas aeruginosa* pili at approximately 95 pN [34]. Beaussart et al. further showed that type IV pili adhesion to hydrophobic surfaces is time-dependent and that individual filaments can withstand forces of up to 250 pN, displaying “constant-force plateau” and “nanospring”-like behaviors relevant to persistence under shear stress [22]. AFM can also distinguish between different pilus types; for instance, type IVc pili of *Caulobacter crescentus* are more adhesive than typical type IVa/b pili due to multiple hydrophobic interactions along the fiber length [35]. These mechanical data provide quantitative insights into the role of TFP in initial colonization during infection. AFM-based force spectroscopy has shown that individual *Pseudomonas aeruginosa* pili can sustain substantial tensile loads and display time-dependent adhesion and force-plateau behaviors relevant to persistence on surfaces exposed to shear stress [22]. Similar approaches have also helped distinguish adhesive differences among pilus classes, including the unusually strong and distributed interactions reported for type IV pili [9,22]. However, AFM studies remain technically demanding because specific pilus engagement depends on careful control of tip functionalization, contact geometry, and surface chemistry, and because force signatures can be difficult to assign unambiguously in cells bearing multiple appendage types [22].

Beyond microscopy, Fourier transform infrared spectroscopy combined with artificial neural network analysis has also been used to estimate piliation levels in a high-throughput manner [36]. Although this approach lacks spatial and dynamic information, it may be useful in industrial or screening contexts.

In recent years, cryo-EM and cryo-ET have enabled near-native structural analysis of type IV pili and their associated assembly machineries (Figure 1) [14]. For example, the *Pseudomonas aeruginosa* PAO1 pilus was resolved at 3.2 Å resolution, revealing the canonical organization of PilA with an N-terminal α-helix forming the filament core and a C-terminal globular region exposed to the environment [19,37]. Similarly, ultrahigh-resolution cryo-EM of enterotoxigenic *Escherichia coli* pili revealed extensive water-mediated interactions that stabilize filament assembly [38]. These structural studies have refined current models of type IV pili assembly and surface interaction, while also providing candidate templates for future inhibitor or antigen design. Cryo-EM and cryo-ET have transformed structural studies of type IV pili by enabling visualization of filaments and assembly machineries in near-native states [9]. Single-particle cryo-EM has resolved purified type IV pili from several organisms at high resolution, revealing conserved features of pilin packing, helical organization, and surface-exposed structural diversity [9,19]. Cryo-EM analysis of pili from other bacteria, including enterotoxigenic *Escherichia coli* and *Thermus thermophilus*, has further shown how hydration, glycosylation, and helical geometry contribute to filament stabilization and specialization [19]. By contrast, cryo-ET is especially informative for intact cells, where it reveals the relative arrangement of the outer membrane secretin, periplasmic components, inner membrane platform, and cytoplasmic ATPases [20,25]. Together, these methods provide a structural bridge between purified filament architecture and the envelope-spanning machinery that supports pilus assembly, extension, and retraction [4,9].

A critical consideration across all visualization approaches is that type IV pili are difficult to identify specifically and to preserve in physiologically relevant states [4,5]. In fluorescence microscopy, nonspecific labeling can generate substantial background, and in many organisms, it remains challenging to distinguish type IV pili from other pili or extracellular appendages without genetic validation [26,39]. Antibody-based detection is not always feasible because suitable reagents are unavailable or because epitope accessibility varies between species and growth conditions [5]. In addition, many bacteria express pili only under restricted environmental conditions that may be difficult to reproduce during microscopy [8]. Similar issues arise in electron microscopy and cryo-EM/cryo-ET, where low contrast, filament flexibility, and structural crowding can complicate confident assignment of type IV pili, especially in intact cells or mixed filament populations [11,20]. In AFM-based studies, selective engagement of a type IV pili with the cantilever tip or substrate often requires extensive optimization, and measured forces can depend strongly on geometry, surface chemistry, and loading regime [22]. These constraints should be considered when comparing results across methods and species.

## 3. Type IV Pili Visualization-Driven Structural Studies

**Figure 2:**
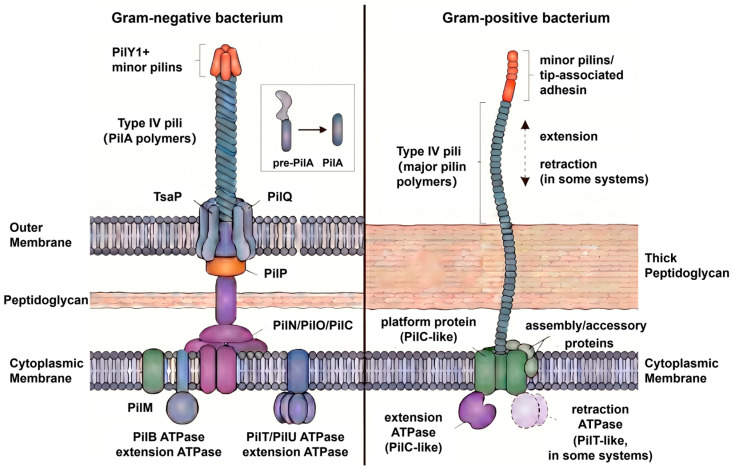
Schematic comparison of type IV pili in Gram-negative and Gram-positive bacteria. In Gram-negative bacteria, type IV pili are assembled from processed PilA subunits and extend through a multi-component envelope-spanning machinery comprising the inner membrane platform, alignment proteins, and the outer membrane secretin PilQ. Pilus extension and retraction are driven by dedicated ATPases [7,25,50,51]. In Gram-positive bacteria, type IV pili are assembled at the cytoplasmic membrane from major pilin subunits, with minor pilins and/or tip-associated adhesins located at the distal end. In the absence of an outer membrane, the pilus traverses the thick peptidoglycan layer, and its assembly is powered by a PilB-like extension ATPase; a PilT-like retraction ATPase is present in some systems. Prepilin processing is illustrated as pre-PilA to mature PilA [52].

The overall architecture of type IV pili is highly conserved, suggesting that these filaments are built on a common structural scaffold despite their broad phylogenetic distribution and functional diversity. Type IV pili are polymerized from major pilin subunits that generally range from 90 to 250 amino acids in length. In most systems, the extended N-terminal α-helices of pilins form a hydrophobic filament core, whereas the C-terminal globular domains remain surface exposed and are therefore positioned to mediate interactions with the extracellular environment [4]. As shown in Figure 2, this basic organization provides a useful framework for understanding how a mechanically stable filament can simultaneously support environmental sensing and surface engagement.

High-resolution structural studies across phylogenetically distant bacteria strongly support this conserved architectural blueprint. Cryo-EM analyses of type IV pili from *Pseudomonas aeruginosa* PAO1 confirmed that PilA adopts the canonical pilin fold and that subunits pack tightly along the filament axis, consistent with earlier observations in the PAK strain [40]. Comparable structural features were subsequently reported for *Streptococcus sanguinis*, further indicating that the type IV pili superfamily shares a common assembly principle [41]. In enterotoxigenic *Escherichia coli*, an ultrahigh-resolution cryo-EM map additionally revealed extensive water-mediated stabilization within and around the filament core, emphasizing that quaternary stability depends not only on pilin packing but also on a finely tuned network of intermolecular interactions [41]. Although the overall architectural logic of type IV pili is highly conserved, functional specialization can arise through modifications superimposed on this shared scaffold [38]. Collectively, these studies suggest that the core architecture of type IV pili is under strong evolutionary constraint, likely because it must support both polymerization and force transmission during pilus extension and retraction.

By contrast, much of the functional specialization of type IV pili appears to arise from structural features superimposed on this conserved core. Variations in filament diameter, helical symmetry, surface topography, and post-translational modification have been documented in multiple species. A particularly informative example comes from *Thermus thermophilus*, which assembles two distinct type IV pili forms with different diameters and helical parameters [14]. The narrow PilA5-based filament contains a pronounced helical groove, whereas the wider PilA4-based filament adopts a more cylindrical architecture. Both are glycosylated, but they differ in the number and position of modified serine residues [14]. Importantly, these differences do not imply unrelated assembly principles. Rather, the two filaments retain a common pilin-based core architecture consistent with the general type IV pili assembly mechanism, while variation in surface-exposed residues and post-translational modifications likely alters intermolecular interactions, filament flexibility, and functional specialization. Thus, type IV pili diversification is better understood as remodeling of a conserved load-bearing scaffold rather than reinvention of the filament core [14,41].

Comparison across Gram-negative and Gram-positive bacteria indicates that the core filament architecture of type IV pili is broadly conserved, but the cellular context in which these filaments are assembled and deployed differs substantially [41]. In Gram-negative bacteria, type IV pili typically traverse an envelope that includes an inner membrane, periplasmic compartment, peptidoglycan layer, and outer membrane secretin [42]. In Gram-positive bacteria, by contrast, pili must be assembled and extruded across a thick peptidoglycan wall in the absence of an outer membrane [42]. Advanced structural and imaging studies suggest that this difference in envelope organization influences pilus localization, extrusion, and substrate access even when the underlying pilin fold and polymerization logic remain similar [41,43,44]. For example, structural work on *Streptococcus sanguinis* supports conservation of the type IV filament blueprint [41], whereas microscopy-based analyses in *Bacillus subtilis* and *Streptococci* emphasize the added importance of cell wall remodeling and broader cell-surface distribution during competence-associated pilus function [43,44,45].

An important limitation of the current structural framework is that most available models describe relatively static states, whereas type IV pili function depends heavily on dynamic behavior. This issue is highlighted by studies of *Neisseria* species. Early docking of pilin crystal structures into low-resolution reconstructions provided an initial model for filament organization [30], but later work showed that rigid-body fitting cannot fully capture the conformational complexity of the assembled pilus [24]. Consistent with this, type IV pili can be stretched to several times their resting length and still recover their original conformation [46]. In *Pseudomonas aeruginosa* and *Neisseria gonorrhoeae*, force-induced structural transitions also expose otherwise hidden epitopes [22,47,48]. Together, these findings indicate that type IV pili should be viewed not simply as static surface appendages, but as mechanically responsive polymers whose structural plasticity is central to motility, adhesion, and host interaction.

Recent advances in cryo-electron tomography have likewise shifted attention from filament architecture alone to the envelope-spanning machinery that controls pilus assembly and remodeling (Figure 2). Structural studies have shown that the adhesin PilY1 plugs the outer membrane secretin channel and connects functionally to the minor pilin complex [49], while analyses of *Thermus thermophilus* revealed that PilQ undergoes open-closed transitions coupled to pilus presence [20]. In intact-cell reconstructions, the relative positioning of the cytoplasmic motor, inner membrane platform, and periplasmic components has further suggested a mechanism in which ATP hydrolysis drives the extraction of pilin subunits from the inner membrane and their incorporation into the growing filament [25]. These findings are important because they directly link structural organization to function: the ability of type IV pili to extend, retract, and bear force depends not only on filament properties but also on the coordination of this trans-envelope nanomachine.

Related studies of homologous motility systems may provide useful mechanistic insight, but must be clearly distinguished from canonical type IV pili machines and interpreted cautiously. For example, cryo-EM structures of GldLM and PorLM—components of the type IX secretion/gliding motility systems in Bacteroidetes—revealed asymmetric membrane-associated motor assemblies in which conserved protonatable residues are required for activity [53]. These systems are not type IV pili; rather, they serve as mechanistically informative homologous surface nanomachines. While these systems are not identical to canonical type IV pili motors, they reinforce the broader principle that surface nanomachines convert electrochemical or chemical energy into mechanical work through highly organized membrane-associated assemblies. For type IV pili, however, the precise coupling between ATP hydrolysis, platform rotation or deformation, and pilin insertion remains incompletely resolved, highlighting an important area for future study [53]. Thus, while lessons from homologous systems illuminate general principles of energy conversion in surface nanomachines, resolving the specific torque-transmission mechanism within the type IV pili motor remains a key challenge for the field.

Current evidence supports a unifying view in which type IV pili function emerges from the interplay of three features: a conserved load-bearing filament core, species-specific variation in surface-exposed architecture, and a dynamic assembly machinery that enables rapid remodeling. This framework helps explain how type IV pili can remain structurally recognizable across bacteria while still being adapted for distinct tasks such as adhesion, twitching motility, DNA uptake, and persistence under mechanical stress. Future work will need to move beyond static descriptions toward integrated models that connect molecular architecture with force-dependent behavior in physiological contexts.

## 4. Type IV Pili Visualization-Driven Functional Studies

### 4.1. Surface Sensing and Twitching Motility

Type IV pili-mediated twitching motility is generally understood as a cyclic process of pilus extension, substrate attachment, and retraction, in which ATP-dependent motor proteins convert chemical energy into mechanical work [54]. In this model, the extension ATPase, typically PilB, promotes pilin polymerization at the pilus base and drives filament extrusion, whereas the retraction ATPase, most commonly PilT, powers pilus depolymerization and generates the tensile force required to pull the cell body forward [4,12]. This framework has provided a unifying mechanistic basis for twitching motility across diverse bacterial species, but its significance extends beyond movement alone: it also establishes type IV pili as dynamic, force-generating nanomachines whose function depends on tight coupling between filament assembly, substrate engagement, and mechanical retraction.

Direct visualization studies have been essential for validating this mechanistic model in living cells. In *Pseudomonas aeruginosa*, total internal reflection fluorescence microscopy of flagellum-deficient cells labeled with an amine-reactive Cy3 dye captured repeated cycles of type IV pili extension and retraction, with comparable rates for both processes and a characteristic short-step, hopping mode of movement [12]. These experiments provided direct evidence that twitching motility is driven by iterative pilus dynamics rather than by passive surface interactions. More broadly, they established live-cell imaging as a critical bridge between genetic models of type IV pili function and the physical behavior of the pilus during surface translocation.

Methodological advances in pilus labeling have progressively improved the spatiotemporal resolution with which type IV pili dynamics can be studied, while also revealing the limitations of earlier approaches. Initial fluorescence-based methods, including immunofluorescence and labeling with bulk fluorescent avidin derivatives, enabled visualization of pilus morphology but interfered with pilus activity and were poorly suited for resolving rapid extension–retraction cycles under native physiological conditions [55,56,57,58,59]. Amine-reactive succinimidyl ester dyes subsequently allowed dynamic tracking of type IV pili in *Pseudomonas aeruginosa*, yet substantial nonspecific labeling generated background fluorescence that limited analytical precision [12]. These limitations underscored a broader point: methods optimized for structural visibility are not necessarily suitable for capturing native pilus behavior, and interpretation of type IV pili dynamics depends strongly on how minimally perturbative the labeling strategy is.

A major improvement came from site-specific pilin labeling based on engineered cysteine substitutions and thiol-reactive maleimide dyes. In *Vibrio cholerae*, introduction of a cysteine residue into the major pilin PilA enabled direct visualization of pili in live cells with substantially improved specificity [18,60]. Importantly, this strategy did more than refine imaging quality; it also enabled mechanistic insight. Using such approaches, studies showed that filament stability is a key determinant of motor-independent pilus retraction, both in systems that encode canonical retraction ATPases, such as competence pili in *Vibrio cholerae* and *Acinetobacter baylyi*, and in systems that lack dedicated retraction motors, such as the toxin-coregulated pilus of *Vibrio cholerae* [60]. These findings broadened the conceptual framework of type IV pili dynamics by indicating that retraction is not always exclusively motor-driven, but can also emerge from intrinsic filament properties.

More recent imaging approaches have further expanded the experimental toolkit and reduced reliance on invasive labeling schemes. Interferometric scattering (iSCAT) microscopy allows label-free visualization of type IV pili dynamics in live cells and is therefore particularly valuable for examining rapid or fragile pilus behaviors that may be altered by fluorophore attachment [13]. In parallel, plasmid-based inducible labeling systems have addressed an important limitation of chromosomal knock-in strategies, which often fail to fully restore wild-type pilus function [32]. In *Pseudomonas aeruginosa* PAO1, inducible expression of a labeled PilA variant allowed controlled incorporation of fluorescently traceable subunits into pili, after which removal of the inducer permitted recovery of the native phenotype through renewed synthesis of wild-type PilA [32]. Beyond improving morphological and dynamic analysis, this approach enabled quantification of subunit turnover within the type IV pili assembly pool and was subsequently extended to *Synechococcus elongatus* PCC 7942, supporting its broader applicability across phylogenetically distinct bacteria [32]. Taken together, these developments illustrate that technical refinement has not merely improved visualization but has directly reshaped mechanistic questions that can be asked about type IV pili assembly, turnover, and function.

Mechanical measurements have provided a complementary perspective by directly linking pilus dynamics to force production. Optical tweezers, for example, permit attachment of retracting pili to micrometer-scale beads and allow quantification of bead displacement under controlled load, thereby providing a direct readout of retraction force [23,61]. Because this approach can also impose external tension on the pilus, it has been particularly informative for probing how type IV pili respond to mechanical resistance and how force influences motor output. Such measurements are critical for moving beyond descriptive imaging toward a physical understanding of how type IV pili drive surface motility under realistic environmental constraints.

Comparative studies across bacterial species further highlight that the conserved extension–attachment–retraction cycle can be adapted to distinct biological contexts. In *Vibrio cholerae*, cysteine-based labeling combined with fluorescence imaging and natural transformation assays demonstrated that pilus filament stability strongly influences competence pilus retraction, directly linking filament material properties to DNA uptake-related function [18,60,62]. In the cyanobacterium *Synechocystis* sp. PCC 6803, by contrast, type IV pili mediate phototactic twitching motility, and directional movement is achieved not through permanent spatial asymmetry in pilus distribution, but through dynamic relocalization of the extension ATPase PilB into a crescent-like domain oriented toward the direction of movement [59,63,64]. These examples emphasize that while the core biophysical logic of type IV pili motility is broadly conserved, functional specialization can arise through differences in filament stability, regulatory polarity, and environmental responsiveness.

Overall, current evidence supports a view of type IV pili motility as an emergent property of three coupled features: ATPase-driven filament dynamics, mechanically competent pili, and regulatory systems that spatially and temporally bias pilus activity. This perspective moves beyond a purely descriptive account of extension and retraction and instead frames type IV pili as adaptable surface nanomachines whose behavior depends on the integration of molecular architecture, mechanical force, and cellular regulation. A remaining challenge for the field is to connect increasingly sophisticated imaging and force measurements with physiologically relevant conditions, so that pilus dynamics can be interpreted not only at the single-filament level but also in the context of collective behaviors, host interaction, and surface colonization.

### 4.2. Biofilm Formation and Surface Adaptation

Type IV pili play central roles in bacterial surface adaptation by coupling surface engagement to regulatory responses that promote the transition from planktonic growth to biofilm-associated states [3,65,66]. Beyond their established functions in adhesion and twitching motility, type IV pili act as sensory appendages that detect physical and chemical cues, including surface contact, mechanical resistance, and local environmental conditions. These inputs are integrated into second messenger pathways, most notably cAMP and cyclic di-GMP (c-di-GMP), allowing pilus activity to influence motility, matrix production, and community development [3,65,66]. Type IV pili-dependent surface adaptation and biofilm initiation have been increasingly understood through visualization methods that connect pilus behavior with intracellular signaling and multicellular organization [39,48]. Rather than relying solely on endpoint biofilm phenotypes, recent studies have used live-cell fluorescence reporters, pilus labeling, and label-free imaging to examine how surface contact, retraction resistance, and second messenger responses unfold in real time [13,15,67]. Type IV pili-dependent regulation is therefore best viewed as a mechanism that links the physical interaction of the cell with its surroundings to long-term adaptive changes in behavior.

This coupling has been studied particularly extensively in *Pseudomonas aeruginosa*. During early surface colonization, type IV pili and adhesins contribute to sensing exopolysaccharide-rich substrates, and resistance to pilus retraction activates the Pil-Chp chemosensory system [65,66]. This promotes cAMP production and activation of the transcription factor Vfr, which regulates genes involved in virulence, pilus-associated functions, and surface behavior [65,66]. These findings support a mechanosensory model in which type IV pili convert retraction-associated resistance into intracellular signaling, thereby enabling cells to distinguish surface-attached from planktonic states. In this context, type IV pili function not simply as adhesive filaments, but as dynamic regulators of surface-responsive gene expression.

Importantly, the signaling consequences of type IV pili engagement are strongly dependent on the physical nature of the interface. Using a three-color fluorescent reporter system to monitor cAMP, c-di-GMP, and a reference signal simultaneously, one study showed that *Pseudomonas aeruginosa* activates distinct second messenger programs at different agarose interfaces [15]. At the agarose–air interface, where flagellar swimming is ineffective, type IV pili-dependent surface sensing preferentially stimulates the cAMP pathway [15]. Impeded pilus retraction is proposed to trigger signaling through PilJ and the adenylate cyclase CyaB, leading to elevated cAMP and Vfr-dependent transcription [15,65]. This response enhances type IV pili-associated motility while repressing flagellar biosynthesis, favoring twitching-based surface exploration under conditions in which flagella contribute little [15]. By contrast, at the agarose–liquid interface, where swimming remains possible, surface-associated envelope stress activates diguanylate cyclases such as WspR, SadC, and SiaD, resulting in c-di-GMP accumulation [15]. Elevated c-di-GMP suppresses motility and promotes production of matrix polysaccharides such as Psl and Pel, thereby driving cells toward sessile growth [15]. These findings are important not only biologically but also methodologically, because they demonstrate how visualization-based reporter systems can resolve heterogeneous and context-dependent signaling states that would be difficult to infer from bulk assays alone [15]. Together, these observations indicate that type IV pili-dependent signaling does not produce a fixed response, but is gated by environmental context and coordinated with the functional state of other motility systems.

This interpretation is reinforced by experiments showing that physical constraints on motility alter signaling output. Increasing medium viscosity with Ficoll reduced swimming speed and decreased the proportion of cells with high c-di-GMP levels, whereas iodixanol, which increases density without substantially affecting viscosity, had little effect [15]. These results suggest that activation of the c-di-GMP program depends less on simple mechanical obstruction than on the motile state of the flagellar apparatus under specific physical conditions. Surface adaptation should therefore be understood as an integrated response in which type IV pili, flagella, and environmental mechanics jointly determine whether cells remain exploratory or commit to biofilm development.

The downstream consequences of these regulatory pathways are evident in early biofilm organization. High c-di-GMP favors matrix synthesis and stabilization of the sessile state, whereas cAMP-associated responses support continued surface exploration [13,65,66]. Consistent with this distinction, perturbations in pilus dynamics can markedly alter surface-associated growth. Fluorescent labeling of pili showed that pilU mutants produce increased numbers of type IV pili but exhibit reduced motility, delayed microcolony formation, and thick multilayered colony edges on semisolid surfaces [68]. These phenotypes indicate that pilus abundance alone is insufficient to promote productive colonization; coordinated extension, retraction, and force transmission are also required. Similarly, changes in type IV pili stability have been linked to both DNA uptake and biofilm-associated behavior [69], further underscoring the close relationship between pilus mechanics and surface adaptation.

Recent high-resolution studies further support this connection between pilus dynamics and biofilm function. Non-invasive interferometric scattering (iSCAT) microscopy showed that expression of a single pilin isoform, PilA2, is sufficient to maintain pilus function, surface attachment, and biofilm formation at levels comparable to the wild type [33]. In *Haloferax volcanii*, iSCAT-based analysis of archaeal type IV filaments under flow conditions showed that simplified pilin composition can still support effective surface attachment and biofilm stability when key dynamic properties are preserved [33]. More broadly, such minimally invasive imaging approaches support the view that biofilm integrity depends not merely on the presence of extracellular filaments, but on the specific mechanical and adhesive behaviors those filaments exhibit under environmental stress [33]. More broadly, type IV pili-mediated interactions with surfaces, and potentially between pili themselves, appear to contribute to biofilm integrity and community organization [33].

Cyanobacteria extend these principles by showing that type IV pili regulation can also be coordinated with temporal environmental cues. In *Synechococcus elongatus* PCC 7942, both pilus morphology and extension–retraction activity fluctuate rhythmically under light–dark cycles [17]. These oscillations correlate strongly with natural transformation efficiency, which is elevated near subjective dusk and generally higher during the dark phase [17]. Under constant light, this rhythmicity gradually disappears, although oscillatory behavior can persist transiently after entrainment [17]. These findings indicate that cyanobacterial type IV pili retain the conserved role of linking external conditions to adaptive behavior, while incorporating an additional layer of circadian regulation that is likely important for the photoautotrophic lifestyle [70].

Current evidence indicates that type IV pili regulate surface adaptation through the integration of mechanical sensing, second messenger signaling, and environmental context. Retraction-associated resistance provides an initial input, cAMP- and c-di-GMP-dependent pathways translate this signal into regulatory outputs, and these outputs determine whether cells continue surface exploration or transition toward matrix production and biofilm formation. Variations in physical environment, interactions with other motility systems, and species-specific regulatory programs further tune this core framework to distinct ecological settings.

### 4.3. Natural Transformation and Horizontal Gene Transfer

**Figure 3:**
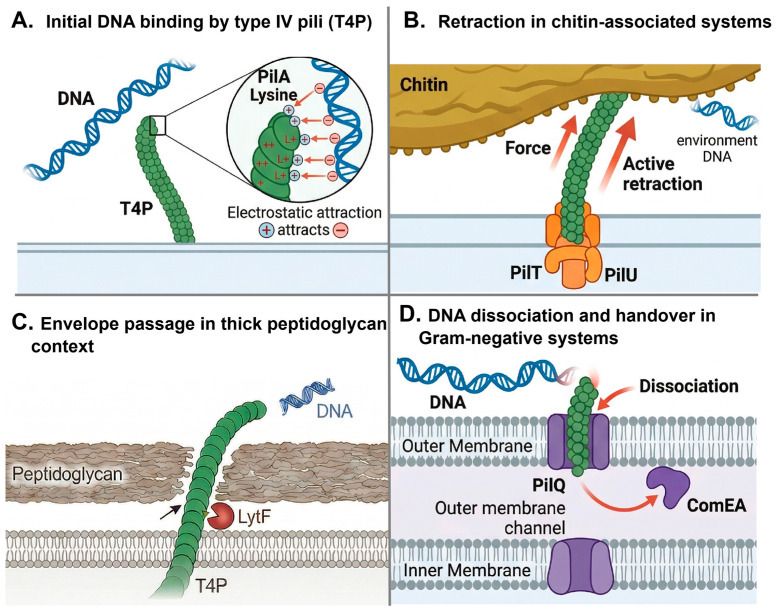
Distinct organism-specific contexts of type IV pilus (type IV pili)-associated extracellular DNA capture and uptake during natural transformation. Panels (**A**–**D**) summarize representative mechanisms reported or proposed in different naturally competent bacteria and are not intended to depict a single universal sequence of events within one organism [73,76]. (**A**) Type IV pili-mediated initial DNA binding may occur through electrostatic interactions between negatively charged extracellular DNA and positively charged residues on pilin subunits, illustrated here by lysine on PilA; in some systems, pilins may also contribute to DNA uptake specificity [77]. (**B**) In chitin-associated competence systems, such as those described in *Vibrio cholerae*, PilT/PilU-driven retraction of a type IV pili-related apparatus may bring extracellular DNA closer to the cell surface and thereby promote uptake initiation [31,56]. (**C**) In thick peptidoglycan contexts, local cell wall remodeling, represented here by LytF, may facilitate pilus emergence and/or DNA access to the competence machinery, as proposed for Gram-positive transformation systems [76]. (**D**) In Gram-negative bacteria, DNA associated with surface appendages may be handed over near the outer membrane to uptake components, including PilQ and ComEA, prior to further trans-envelope transport [73,78].

Type IV pili constitute a major machinery for natural transformation in many bacteria by coupling extracellular DNA capture to its delivery across the cell envelope. As summarized in Figure 3, this process generally involves a stepwise sequence of pilus extension, substrate recognition, DNA binding, retraction-driven retrieval to the cell surface, and handover to competence-associated transport proteins. Although the molecular details differ across species, this framework provides a useful basis for comparing how type IV pili-mediated DNA uptake is adapted to different ecological and cell-envelope contexts. Rather than acting as passive surface appendages, these filaments function as dynamic uptake devices whose activities depend on coordinated extension, substrate engagement, retraction, and transfer to downstream competence proteins [71,72,73]. This multistep process places type IV pili at the center of horizontal gene transfer, while also highlighting that transformation efficiency is determined not simply by pilus presence, but by how effectively pilus dynamics, DNA recognition, and envelope translocation are integrated.

A key level of regulation lies in how type IV pili recognize extracellular DNA. In some species, DNA uptake is highly selective. In *Neisseria gonorrhoeae*, the minor pilin ComP mediates sequence-specific recognition of a 10–12 bp DNA uptake sequence, thereby biasing transformation toward closely related DNA and promoting preferential acquisition of homologous genetic material [71]. By contrast, other systems appear to rely on broader, sequence-independent binding strategies. In *Pseudomonas aeruginosa*, the C-terminal β-sheet region of the major pilin PilA contains multiple lysine residues that generate a positively charged surface capable of interacting electrostatically with the negatively charged DNA backbone [72]. Atomic force microscopy has provided direct evidence for this pilus–DNA association [72]. Considered together, these examples indicate that type IV pili do not mediate a single universal mode of DNA capture; rather, they support a continuum ranging from highly discriminatory uptake to more permissive DNA binding, depending on the ecological and evolutionary context of the organism.

Following DNA recognition, pilus dynamics determine whether bound DNA can be productively brought to the cell surface for transport. Studies in naturally competent vibrios have shown that transformation pili can extend to several micrometers, contact extracellular DNA, and then retract to pull the substrate toward the cell envelope, where proteins such as ComEA are thought to mediate subsequent internalization [31,74]. Similar behavior has been observed in *Streptococcus pneumoniae*, in which competence pili are highly dynamic and undergo repeated cycles of extension and retraction during DNA uptake [45]. Wide-field fluorescence microscopy with labeled DNA has been particularly informative in establishing this model, because it directly links pilus motion to substrate capture and delivery [31]. Importantly, mutants defective in DNA binding display markedly reduced transformation efficiency, supporting the conclusion that physical engagement of DNA by the pilus is not incidental, but an essential early step in the uptake pathway [31,74].

The efficiency of type IV pili-mediated transformation also depends on the mechanical demands imposed by the environment. On solid chitinous surfaces, for example, DNA may be less freely accessible than in liquid environments, requiring stronger pilus-mediated pulling forces for successful uptake. Under these conditions, the auxiliary retraction ATPase PilU becomes critical: quantitative transformation assays have shown that pilU mutants are severely impaired, or nearly abolished, in their ability to acquire DNA from chitin surfaces [75]. This finding suggests that high-force retraction is not merely an accessory feature of type IV pili function, but can become physiologically indispensable when DNA must be detached from structured substrates. More broadly, it emphasizes that natural transformation should be understood as a mechanically contingent process, in which the success of DNA acquisition depends on both pilus dynamics and the physical properties of the surrounding habitat.

In Gram-positive bacteria, additional constraints arise from the cell wall, making DNA uptake dependent not only on pilus function but also on envelope remodeling. In *Bacillus subtilis*, competence pili are distributed along the long axis of the cell rather than being restricted to the poles, indicating that initial DNA capture can occur over a broader cell surface area [44]. This spatial pattern has been visualized using fluorophore-coupled maleimide labeling combined with epifluorescence microscopy [44]. Genetic analyses further show that positively charged residues in the minor pilin ComGF contribute substantially to DNA uptake, consistent with a direct role in substrate interaction [79]. At the same time, efficient pilus extrusion through the thick peptidoglycan layer appears to require the cell wall hydrolase LytF. Immunoblotting showed that deletion of lytF markedly reduced extracellular ComGC levels, while immunofluorescence microscopy using FLAG-tagged ComGC directly visualized a loss of surface-exposed pili in the lytF mutant. Together, these observations support a model in which LytF-mediated local peptidoglycan remodeling enables pilus emergence and thereby promotes transformation [80]. These observations underscore that, particularly in monoderm bacteria, type IV pili-dependent DNA uptake cannot be fully understood without considering the structural constraints of the cell envelope.

Current evidence supports a stepwise but species-tuned model of type IV pili-mediated natural transformation (Figure 3): pili first recognize extracellular DNA through either sequence-specific or electrostatic interactions, then retrieve it through dynamic retraction, and finally deliver it to envelope-associated transport components for translocation into the cell [31,44,71,72,74,75,79,80]. The precise implementation of this pathway varies across bacteria according to DNA recognition strategy, retraction force requirements, and envelope architecture. Framed in this way, type IV pili are not simply DNA-binding filaments, but adaptable molecular devices that integrate substrate recognition, mechanical work, and cell envelope traversal to control horizontal gene transfer.

## 5. Technological Applications and Future Perspectives

**Figure 4:**
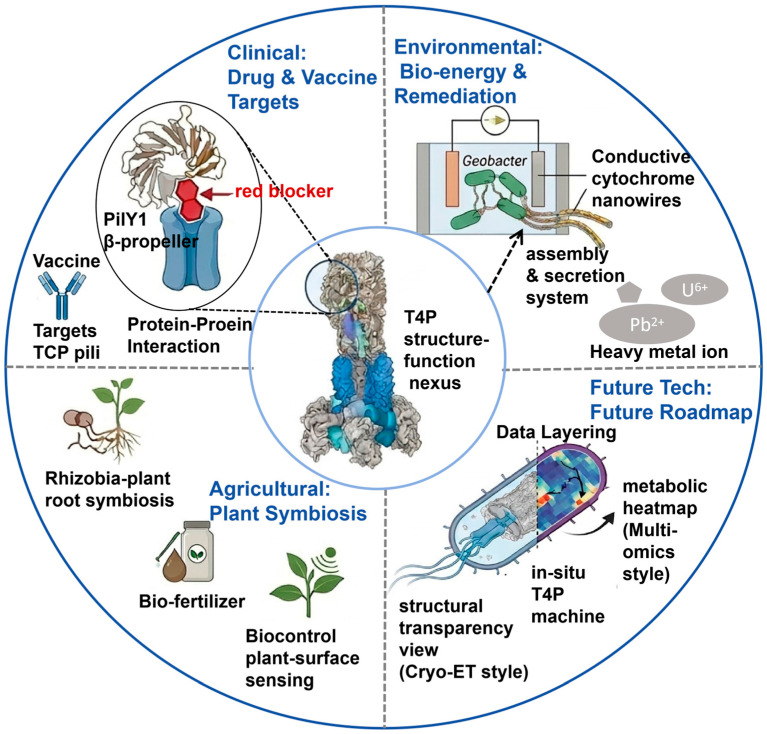
Technological applications and future perspectives enabled by visualization of type IV pili (T4P). Clinical applications: Structural and imaging studies identify candidate antivirulence targets and vaccine-relevant features, including adhesin-associated regulatory interfaces, assembly surfaces, and surface-exposed regions. The PilY1-centered module exemplifies a potential therapeutic target. Environmental biotechnology: visualization helps distinguish canonical type IV pili from other extracellular conductive appendages, including cytochrome-based nanowires in Geobacter, refining models of extracellular electron transfer relevant to bioenergy and bioremediation. Agricultural and microbial engineering: imaging-informed phenotyping supports studies of surface colonization, plant-associated persistence, symbiosis, and biofilm organization, with potential applications in microbial engineering and agriculture. Future perspectives: progress will depend on correlative, multimodal approaches integrating cryo-ET, live-cell fluorescence microscopy, iSCAT, force-based methods, genetics, and omics analyses to link type IV pili architecture, dynamics, and function in complex environments.

Visualization-driven advances in type IV pili biology have implications beyond basic mechanisms, although most applications remain at an early stage. Rather than providing ready-to-use solutions, current imaging and structural studies mainly identify candidate molecular interfaces, mechanistic vulnerabilities, and design principles that may support future intervention or engineering (Figure 4) [4,9].

In anti-infective research, type IV pili and their associated regulatory modules are attractive antivirulence targets because they contribute to motility, adhesion, DNA uptake, and biofilm-associated persistence [4,49,81]. Imaging and structural studies have been particularly useful for identifying functionally important interfaces within the pilus machine, such as adhesin-associated regulatory nodes (e.g., the PilY1-PilQ interaction in *Pseudomonas aeruginosa* [35]), filament assembly surfaces, or components required for force transmission, that may be vulnerable to antivirulence intervention [49]. However, translation into clinically useful inhibitors remains challenging because issues such as target accessibility, specificity, and pathway redundancy are still unresolved. In vaccine-related contexts, high-resolution structural information can assist epitope mapping by identifying surface-exposed and conformationally constrained pilin regions, although antigenic variability and limited accessibility remain major obstacles [82,83]. Type IV pili-related structures may also inform vaccine design in selected pathogens. High-resolution analysis can help identify surface-exposed or conformationally constrained regions with potential antigenic value. However, antigenic variation, structural heterogeneity, and limited epitope accessibility may reduce the feasibility of broadly effective pilus-based vaccines. Thus, visualization currently contributes more to epitope mapping and antigen selection than to validated vaccine development [39,84]. In environmental microbiology and biotechnology, visualization has helped clarify the identity and function of extracellular appendages. A notable example is *Geobacter sulfurreducens*, in which structural work helped distinguish canonical type IV pili from cytochrome-based conductive filaments, a distinction that is important for bioelectrochemical engineering, as accurate filament identification is necessary for rationally optimizing extracellular electron transfer in systems relevant to bioremediation and microbial fuel cells [85,86]. Furthermore, visualization has supported studies of surface colonization and microbial community organization. It has also enabled imaging-informed phenotyping for engineering of microbial colonization or plant-associated persistence [4,66,87,88]. From a technical and methodological perspective, future progress will depend on integrating complementary approaches. Cryo-ET is well suited for resolving native machine architecture, whereas fluorescence microscopy and iSCAT reveal dynamic behaviors in live cells [13,45], and AFM or optical trapping quantify the associated mechanical outputs [22]. Correlative and multimodal workflows that connect structural states, dynamics, and physiological function will be especially valuable [4,9]. Combining visualization with genetics, transcriptomics, single-cell signaling analyses, and omics-based approaches should provide a more complete picture of how type IV pili are coordinated with motility, competence, biofilm formation, and collective behaviors in complex environments.

The long-term value of type IV pili visualization lies not only in describing pili but also in helping explain when, where, and how specific structural and dynamic states contribute to microbial behavior in complex environments.

## 6. Conclusions

Visualization research on microbial type IV pili has progressed from static structural description to a new stage of dynamic mechanism elucidation. Breakthroughs in cryo-electron microscopy, super-resolution fluorescence imaging, and interferometric scattering microscopy have enabled researchers to reveal, in situ and from nanoscale to atomic resolution, the dynamic regulatory mechanisms of type IV pili in twitching motility, surface sensing, DNA uptake, and biofilm formation. These findings not only deepen our understanding of the structural diversity, functional complexity, and environmental adaptation strategies of type IV pili but also provide theoretical foundations and technological targets for anti-infective drug design, vaccine development, environmental remediation, and agricultural biocontrol. In the future, the deep integration of multimodal imaging technologies with multi-omics will further elucidate the synergistic mechanisms between type IV pili and the cell cycle as well as metabolic networks, fully unleashing their scientific value and application potential.

## Data Availability

No new data were created or analyzed in this study. Data sharing is not applicable to this article.
